# Ideal Local Anesthetic for Intraperitoneal Gallbladder Bed Infiltration Following Laparoscopic Cholecystectomy: A Randomized Controlled Trial

**DOI:** 10.7759/cureus.71122

**Published:** 2024-10-09

**Authors:** Syed M Ahmed, Sidra Shabbir, Nauman A Rana, Atia Khatoon, Umar F Ghani, Irmaghana Basharat, Muhammad N Khan, Fahd M Hameed, Muhammad F Dar

**Affiliations:** 1 General Surgery, Pakistan Air Force Hospital, Islamabad, PAK

**Keywords:** intraperitoneal bupivacaine, intraperitoneal lignocaine, laparoscopic cholecystectomy, perioperative pain management, visual analog scale

## Abstract

Background

Laparoscopic cholecystectomy is the gold standard operation for symptomatic cholelithiasis; however, pain remains a major factor in increasing morbidity and length of hospital stay. Infiltration of the gallbladder bed with a local anesthetic has been shown to improve postoperative pain after laparoscopic cholecystectomy, although it is unclear which local anesthetic provides superior pain relief.

Objective

The aim of this study was to compare the efficacy of various local anesthetics on postoperative pain when instilled intraperitoneally in the gallbladder bed following laparoscopic cholecystectomy.

Methods

Patients undergoing laparoscopic cholecystectomies were randomized into three groups of 30 patients each, depending on the local anesthetic instilled in the gallbladder bed: lignocaine (Group A), bupivacaine (Group B), or a combination of lignocaine and bupivacaine (Group C). Pain was measured using the Visual Analogue Scale (VAS) at two, six, 12, and 24 hours following surgery. Secondary outcomes included vitals, rescue analgesic use, return of bowel function, time to ambulation, and presence of nausea.

Results

Group C showed the lowest pain scores at all time points compared to the other groups (p < 0.05). Mean arterial pressures and heart rates were lower in Group C than in Group A at two and 24 hours. Return of bowel function and time to ambulation was earlier in Group C than in Group B. No significant complications were noted in any of the groups.

Conclusion

We conclude that a combination of lignocaine and bupivacaine instillation in the gallbladder bed provides the most effective pain relief following laparoscopic cholecystectomy, without significant complications at the doses used.

## Introduction

In the Western world, gallstones affect 10%-20% of the people [1]. It is estimated that around 20% of people with gallstones become symptomatic, manifesting as either biliary colic, acute cholecystitis, obstructive jaundice, or gallstone pancreatitis [2]. For symptomatic gallstones, the preferred treatment is cholecystectomy [3], which is now frequently done laparoscopically, with trends shifting to robotic surgery. Every year, 500,000 cholecystectomies are done in the United States and around 66,660 in the UK [4] alone, with around 90% of these being performed laparoscopically [5]. Not only is the number of cholecystectomies increasing every year but so is the proportion of cholecystectomies being performed laparoscopically [6].

One of the major advantages of laparoscopic cholecystectomies is lesser postoperative pain when compared to open surgery. However, pain is still an important factor in increasing morbidity after the procedure and lengthening hospital stay [7, 8]. Pain could be either somatic from the incisional sites where the ports are inserted, or it could be visceral from the tissue injury during dissection and removal of the gallbladder from the surrounding structures [9]. Pre/post-incisional instillation of the local anesthetic at the sites of port insertion is a known way of reducing somatic pain [10]. For visceral pain, local anesthetic infiltration of the gallbladder bed after removal of the gallbladder has been shown to improve postoperative pain and reduce the incidence of rescue analgesic use [4, 9, 11-16]. However, the best agent for this intraperitoneal instillation and its dose has not yet been identified, with contradictions in the results [17, 18].

In our center, we use a combination of bupivacaine and lignocaine, which has shown improvement in patient satisfaction. However, we do not know if it is superior to either of the agents used in isolation. To the author's knowledge, there are no current published studies that compare the combination of these drugs used in isolation when used intraperitoneally for any open or laparoscopic cholecystectomies.

The aim of this study was to compare the analgesic effects of different local anesthetics when used intraperitoneally in a gallbladder bed on visceral pain following laparoscopic cholecystectomy. We hypothesized that a combination of lignocaine and bupivacaine would provide better analgesia following laparoscopic cholecystectomy than when either of the agents is used in isolation. 

## Materials and methods

This was a double-blinded, randomized controlled trial conducted at Pakistan Air Force Hospital (PAF), a tertiary care hospital in Islamabad, Pakistan, from August 2023 to July 2024. Approval was obtained from the institution’s ethical committee (approval number: SGR-2021-137-2499-1) and was performed in accordance with the ethical standards laid down in the 1964 Declaration of Helsinki and its later amendments. The study was registered on clinicaltrials.gov (NCT06605235). Informed consent was obtained from 90 patients undergoing laparoscopic cholecystectomy (elective and urgent) between the ages of 18 and 75 years. The primary pathologies for the subjects included symptomatic cholelithiasis, acute/chronic/recurrent cholecystitis, biliary pancreatitis, interval cholecystectomy for necrotizing pancreatitis, and symptomatic gallbladder polyps. Patients were either under American Society of Anesthesiologists (ASA) categories I or II. Experienced laparoscopic surgeons who performed the procedure on a regular basis performed standard four-port laparoscopic cholecystectomy. Exclusion criteria included patients on chronic analgesics, those who had received pain medications 24 hours immediately preceding the procedure, intraoperative drain placement, common bile duct (CBD) exploration or T-tube placement, BMI >40 kg/m^2^, and those patients who were allergic to or could otherwise not be administered the medications being tested.

The sample size was calculated using the WHO calculator, for which the anticipated population mean was 0.92 and the test value of the population mean was 2.08 [17]. With the population standard deviation at 1.28, the level of significance taken at 5%, and the power of the test taken at 90%, a total sample size that would show a significant difference in pain scores was calculated to be 90 (30 in each of the three groups).

Patients were randomly assigned to one of three groups using simple randomization with computer-generated numbers. In Group A, patients received 20 ml of 1% lignocaine (xyloaid-lignocaine hydrochloride (HCl) injection) infiltrated in the gallbladder bed after removal of the gall bladder, along with 10 ml of 1% lignocaine infiltration at the port sites at the end of the procedure. Subjects in Group B received 20 ml of 0.25% bupivacaine (bupicain-bupivacaine HCl injection) infiltration in the gallbladder bed, along with 10 ml of 0.25% bupivacaine at port sites. In Group C, patients received a mixture of 10 ml of 1% lignocaine along with 10 ml of 0.25% bupivacaine infiltrated in the gallbladder bed, followed by 5 ml of 1% lignocaine along with 5 ml of 0.25% bupivacaine at the port sites.

All patients received standard medications, including antibiotics, proton pump inhibitors (PPIs), and one analgesic (injection of ketorolac 30 mg intravenously every eight hours). House surgeons blinded to the study groups collected postoperative data on a standardized proforma, including the Visual Analogue Scale (VAS) for pain at two, six, 12, and 24 hours post-procedure. If the patient’s pain score was more than five and not settled with ketorolac, then rescue analgesia (injection of nalbuphine 5 mg intravenously) was used, and the timing was noted on the proforma. At each of these time points, the patient's vitals were also recorded, along with whether the patient was experiencing any nausea or vomiting if they had started passing flatus or started ambulating, and whether they had shoulder tip pain.

Statistical analysis was performed using analysis of variance (ANOVA) on IBM SPSS Statistics software, version 29 (IBM Corp., Armonk, NY). A p-value of ≤ 0.05 was considered to be significant. The datasets generated during and/or analyzed during the study are available from the corresponding author upon reasonable request.

## Results

Demographic data

The demographics were similar across all three groups (Table 1).

**Table 1:** Patient demographics SEM: standard error of mean

Characteristics	Group A	Group B	Group C
Age in years (Mean ± SEM)	42.2 ± 2.671	43.2 ± 2.524	43.9 ± 2.473
Male-to-Female ratio	7:23	6:24	7:23
Hospital stay (days)	1	1	1

The mean age in Group A was 42.2 years (SEM 2.671) with a male-to-female ratio of 7:23. In Group B, the average age was 43.2 years (SEM 2.524), with female predominance in the sample (male-to-female ratio of 6:24). In Group C, the mean age was 43.9 years with an SEM of 2.473 and a male-to-female ratio of 7:23.

Postoperative pain

**Figure 1:**
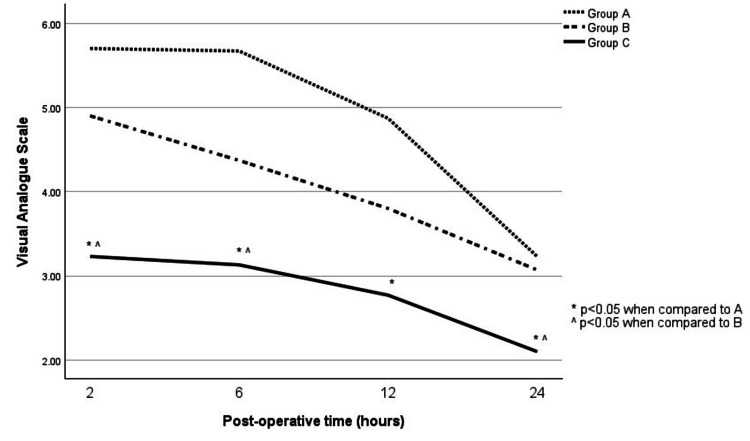
Pain score (as measured by the visual analogue scale) at various time points postoperatively Statistical test used: ANOVA (p ≤ 0.05 is considered significant)

When comparing postoperative pain (as assessed by VAS), Group C showed the best analgesic effects (Figure 1).

At postoperative hours two, six, 12 and 24, patients in Group C (mean VAS scores of 3.23±0.321, 3.13±0.338, 2.77±0.257, 2.10±0.273) showed a statistically significant lower pain score than Group A (mean VAS scores of 5.70±0.448, 5.67±0.372, 4.87±0.398, 3.23±0.266; p<0.01). Group C also showed significantly improved scores than Group B at time points two, six, and 24 hours postoperatively (mean VAS scores of 4.90±0.312, 4.37±0.294, 3.07±0209; p<0.05). At 12 hours postoperatively, Group C had lower pain scores than Group B (mean VAS score 3.80±0.334), but not statistically significant (p = 0.095) (Table 2, 3).

**Table 2:** Pain scores (as measured by the VAS) and time taken for resolution of shoulder tip pain VAS: visual analogue scale; SEM: standard error of mean

Group	VAS score (Mean ± SEM)				Shoulder tip pain resolution in hours (Mean ± SEM)
	2 hours	6 hours	12 hours	24 hours	
A	5.70 ± 0.448	5.67 ± 0.372	4.87 ± 0.398	3.23 ± 0.266	2.40 ± 1.337
B	4.90 ± 0.312	4.37 ± 0.294	3.80 ± 1.827	3.07 ± 0.209	2.60 ± 1.210
C	3.23 ± 0.321	3.13 ± 0.338	2.77 ± 0.257	2.10 ± 0.273	2.40 ± 1.337

**Table 3:** Statistical analysis of pain scores between study groups Statistical test used: ANOVA (p ≤ 0.05 is considered significant)

Postoperative time (hours)	Groups A-C (p-value)	Groups B-C (p-value)	Groups A-B (p-value)
2	< 0.001	0.005	0.375
6	< 0.001	0.033	0.023
12	< 0.001	0.095	0.080
24	0.006	0.023	1.000

There was no difference regarding shoulder tip pain between all three groups, with the mean time for disappearance of shoulder tip pain being 2.4, 2.6, and 2.4 hours for Groups A, B, and C, respectively (Table 2).

Rescue analgesic requirement

Four patients in Group A required rescue analgesia, whereas two patients required injection of nalbuphine in Group B, and only one patient in Group C. Of the four patients in Group A, they required rescue analgesic at five, six, and eight hours post-procedure, respectively. In Group B, the patients received nalbuphine at one and six hours post-procedure. In Group C, the patient got the medication six hours postoperatively.

Vital signs

Mean arterial pressures were calculated from the systolic and diastolic blood pressures of the patients. The mean arterial pressures of subjects in Group C were significantly lower than subjects in Group A at two and 24 hours (Table 4).

**Table 4:** Comparison of vital signs among the study groups Statistical test used: ANOVA (p ≤ 0.05 is considered significant); * p<0.05 when compared to Group A SEM: standard error of mean

Postoperative time (hours)	Mean arterial pressures (Mean ± SEM)			Heart rate (Mean ± SEM)			Respiratory rate (Mean ± SEM)		
	A	B	C	A	B	C	A	B	C
2	93.47±2.14	89.73±1.76	86.33±1.79^*^	83.13±1.73	77.83±2.23	74.03±2.21^*^	16.90±0.55	15.90±0.71	16.63±0.41
6	87.80±1.04	90.33±1.27	90.73±1.15	82.60±1.42	78.60±2.45	78.60±1.91	15.87±0.47	15.43±0.58	16.77±0.34
12	87.47±1.15	90.23±0.92	88.47±1.35	82.10±1.86	86.23±1.84	81.30±1.49	16.30±0.37	15.97±0.64	16.37±0.43
24	95.23±1.69	89.90±1.63	88.47±1.35^*^	78.90±1.29	77.33±0.82	80.87±1.59	14.67±0.46	15.63±0.58	16.43±0.34

However, the groups did not show any significant difference at other time points. A similar pattern was seen in the heart rates of the patients in Groups A and C, with lower heart rates in Group C as compared to Group A at only two hours post-procedure (Table 4). None of the other vital signs showed any significant differences.

Other secondary outcomes

**Figure 2:**
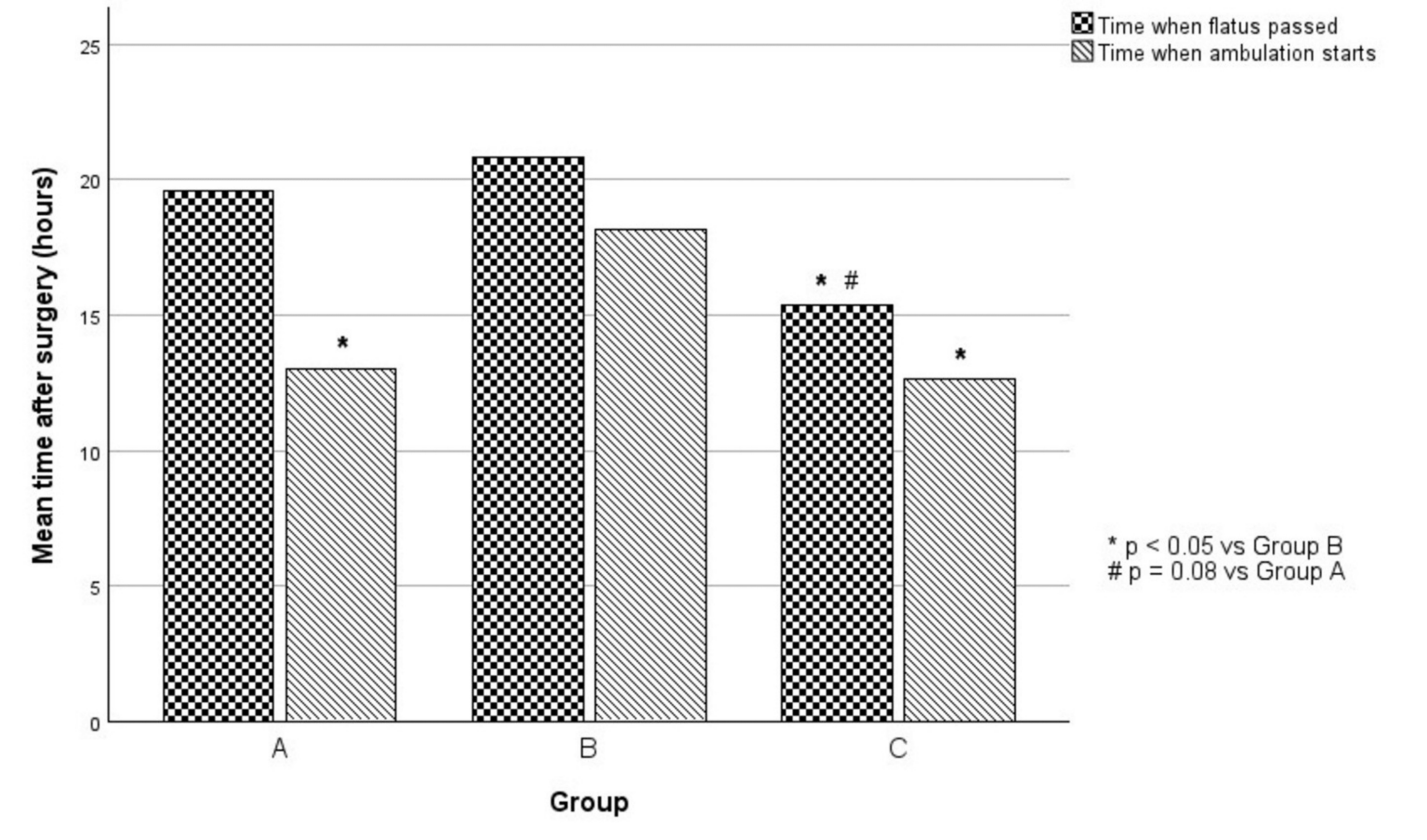
Mean time for patients to start ambulation and return of bowel function Statistical test used: ANOVA (p ≤ 0.05 is considered significant)

Subjects in Groups A and C started ambulating earlier than subjects in Group B (p<0.05) (Figure 2).

Postoperative nausea did not show any difference between the groups. Looking at the time to pass flatus, patients in Group C passed flatus earlier than patients in both Groups A (p = 0.082) and B (p = 0.015).

Complications

None of the patients in the study had any complications and were discharged on postoperative day one.

## Discussion

A few of the major advantages of laparoscopic surgery are reduced postoperative pain, shorter hospital length of stay, and early return to work. However, pain control, even after laparoscopic surgeries, is a cause of morbidity in patients, resulting in delayed hospital discharges and reduced patient satisfaction [17]. Multiple factors affect postoperative pain following laparoscopic cholecystectomy, including retained carbon dioxide gas in the abdominal cavity irritating the diaphragm, somatic pain from port site incisions, and dissection of tissues, including the gallbladder, from the gallbladder bed [9].

To improve postoperative pain control, different modalities have been used and well established, including port-site and intraperitoneal local anesthesia (LA) infiltration. Various anesthetic agents have been used, including bupivacaine, lignocaine, levobupivacaine, and ropivacaine, and most of these studies have shown a decrease in postoperative pain scores from instillation of these LA agents [9, 11, 12, 14, 17-25]. However, much debate exists as to which agent provides the optimum pain control, with some studies leaning towards the idea that there is no difference.

Lignocaine is a short-acting monocarboxylic acid amide with an average duration of action of one to two hours, with onset of action in less than two minutes [26]. In contrast, bupivacaine is a long-acting amide with a slow onset of up to five minutes but a longer duration lasting from two to four hours [26]. A combination of these two agents has been shown to be an ideal local anesthetic in various settings, bringing together the rapid onset of lignocaine with the longer duration of bupivacaine. It has been the LA of choice at the Lichtenstein Hernia Institute for performing open meshplasty for primary reducible inguinal hernias [27]. It has even been shown to reduce rescue analgesic requirements if used as a combination in caudal blocks following circumcision as compared to bupivacaine alone [28].

Our study confirmed these results: a combination of lignocaine and bupivacaine resulted in lower pain scores when instilled intraperitoneally in the gallbladder bed following laparoscopic cholecystectomy when compared to either of the drugs used in isolation. This effect was significant starting from as early as two hours up to 24 hours post-procedure. The prolonged analgesic effects seen in this study could be partly explained due to the slowed intraperitoneal absorption of the agents [17]. The improved analgesic effect of the combination is reinforced by the fact that only one patient required rescue analgesia in this group as compared to four in the lignocaine-only group; however, this difference was not significant.

Looking at the vital signs, the mean arterial pressures in Group C were significantly lower than Group A at two and 24 hours postoperatively, along with a lower heart rate in the combination group as compared to lignocaine alone at two hours postoperatively. This could indicate improved pain control at these time points, but this inference is hard to make as many of these patients had hypertension with variable control, and some were even on beta blockers, which was not catered for in the data analysis. The rest of the vital signs were comparable in all three groups, reinforcing the safety of these drugs when used intraperitoneally, as shown in previous studies [4, 9, 11, 12, 14-18, 20-22, 24, 29, 30].

Postoperative nausea, time to ambulation, and time to pass flatus are other factors that affect the length of hospital stay and thus overall cost. Postoperative nausea did not show any difference among the groups. However, patients in the combination group and the lignocaine-alone group started ambulation significantly earlier than the patients who just received bupivacaine. This could possibly be attributed to the faster onset of action of lignocaine used in both Groups A and C. Return of bowel function, as indicated by passing of flatus, was earlier in patients of the combination group significantly when compared to bupivacaine alone, and near-significant when compared to the lignocaine-only group. This could again be related to the fact that bowel function following surgery is affected by factors like severe pain and immobility, among others. Therefore, as pain is well controlled and patients are mobilizing in the combination group earlier, the gut motility returns faster.

There are a few limitations of this study. We only looked at postoperative pain in the first 24 hours following the procedure and did not look at the effect that these drugs have on long-term pain control. Secondly, we looked at vital signs among other secondary outcomes; however, we did not include patient satisfaction and quality of recovery. We also did not cater to the individual BMI of each patient and gave a standard drug dose to all the participants. Another limitation was that we did not monitor pain scores at rest and with activity separately, which would have better elucidated the analgesic effects of the drugs we were studying. Future studies should focus on weight-dependent dosing for the patients for an ideal analgesic effect and have a longer follow-up to assess the effect of this intervention on chronic pain following the procedure. 

## Conclusions

We conclude that following laparoscopic cholecystectomy, gallbladder bed infiltration with a combination of lignocaine and bupivacaine has improved analgesic effects lasting up to at least 24 hours following surgery when compared to either of the agents used alone. This combination is safe at the doses used in the study and does not result in any significant complications. The formulation is associated with an earlier return of bowel function and early ambulation. Further studies are recommended to validate the ideal dose and superiority of this formulation in other surgical procedures as well.
